# A robust deep learning approach for tomato plant leaf disease localization and classification

**DOI:** 10.1038/s41598-022-21498-5

**Published:** 2022-11-03

**Authors:** Marriam Nawaz, Tahira Nazir, Ali Javed, Momina Masood, Junaid Rashid, Jungeun Kim, Amir Hussain

**Affiliations:** 1 Department of Computer Science, University of Engineering and Technology Taxila, Taxila, 47050 Pakistan; 2 Faculty of Computing, Riphah International University, Islamabad, Pakistan; 3 Department of Software Engineering, University of Engineering and Technology Taxila, Taxila, 47050 Pakistan; 4 Department of Computer Science and Engineering, Kongju National University, Cheonan, 31080 South Korea; 5 Department of Software, Kongju National University, Cheonan, 31080 South Korea; 6 Centre of AI and Data Science, Edinburgh Napier University, Edinburgh, EH11 4DY UK

**Keywords:** Plant sciences, Diseases, Mathematics and computing

## Abstract

Tomato plants' disease detection and classification at the earliest stage can save the farmers from expensive crop sprays and can assist in increasing the food quantity. Although, extensive work has been presented by the researcher for the tomato plant disease classification, however, the timely localization and identification of various tomato leaf diseases is a complex job as a consequence of the huge similarity among the healthy and affected portion of plant leaves. Furthermore, the low contrast information between the background and foreground of the suspected sample has further complicated the plant leaf disease detection process. To deal with the aforementioned challenges, we have presented a robust deep learning (DL)-based approach namely ResNet-34-based Faster-RCNN for tomato plant leaf disease classification. The proposed method includes three basic steps. Firstly, we generate the annotations of the suspected images to specify the region of interest (RoI). In the next step, we have introduced ResNet-34 along with Convolutional Block Attention Module (CBAM) as a feature extractor module of Faster-RCNN to extract the deep key points. Finally, the calculated features are utilized for the Faster-RCNN model training to locate and categorize the numerous tomato plant leaf anomalies. We tested the presented work on an accessible standard database, the PlantVillage Kaggle dataset. More specifically, we have obtained the mAP and accuracy values of 0.981, and 99.97% respectively along with the test time of 0.23 s. Both qualitative and quantitative results confirm that the presented solution is robust to the detection of plant leaf disease and can replace the manual systems. Moreover, the proposed method shows a low-cost solution to tomato leaf disease classification which is robust to several image transformations like the variations in the size, color, and orientation of the leaf diseased portion. Furthermore, the framework can locate the affected plant leaves under the occurrence of blurring, noise, chrominance, and brightness variations. We have confirmed through the reported results that our approach is robust to several tomato leaf diseases classification under the varying image capturing conditions. In the future, we plan to extend our approach to apply it to other parts of plants as well.

## Introduction

According to a prediction by the Food and Agriculture Organization (FAO) of the United Nations, the world population could reach 9.1 billion by 2050, ultimately increasing food requirements^1^. Whereas the unavailability of large cultivation space and shortage of clean water is the major hindrance to the progression of food quantity. The need of the hour is to increase the growth rate of food in small spaces to cater to the needs of a large population. However, the presence of several plant diseases also causes a significant reduction in both food quality and quantity. Therefore, it is necessary to diagnose these plant abnormalities in the initial phase as these damages have a bad impact on the agriculturalist’s revenue and can also increase food prices. Its more devastating form can introduce starvation situations, especially in poor countries. Mostly, humans performed crop inspections by visiting the cultivation areas. However, visual inspection is a tedious task that is highly dependent on the availability of human resources. Secondly, human crop inspection is not much reliable as it is difficult for them to view individual plants^2^. Therefore, the identification and categorization of crop diseases at the early stage is a mandatory task for improving the food growth rate and saving the agriculturalists from expensive spray procedures. Therefore, to avoid manual inspection, researchers are designing automated systems for crop disease recognition^3^.

Although there exist a variety of crop plants, including Potato, Onions, Strawberry, Cheery, etc., however, the Tomato plant has the highest consumption rate with the range of 15 kg per capita in a year and counts for about 15% of the total vegetable consumption in all around the globe^4^. Therefore, the identification and classification of various tomato leaf diseases are the main focus of this study. Among the world's vegetable crop production, tomatoes rank first with an output of more than 170 million tons annually^5^. Various countries produce tomatoes globally each year, with India, Turkey, Egypt, and the United States being the leading producers^6^. According to a study conducted by the FOA, the major hindrance to the production rate of tomatoes is the presence of several diseases, where most of which start from the tomato plant leaves and results in to decrease in the production quantity from 8 to 10% every year^7^. Computer-aided systems can assist the practitioners in better identifying the tomato leaf diseases at the earliest stages to save the farmers from extensive losses. At first, the research community employed the approaches of molecular biology and immunology areas for tomato leaf disease inspection^8,9^. However, these works are suffering from high processing complexity and need a lot of human expertise. Mostly, crop cultivation is performed by poor people having a low source of income^10^, so, such expensive solutions are not feasible for them^11^. With the progression of machine learning (ML), researchers have applied several hand-crafted techniques in agricultural science^12^. The easier digital data acquisition methods have assisted to collect extensive data in real-time which is utilized by the ML-based methods to make intelligent decisions. Such approaches namely support vector machines (SVM)^13^, decision trees (DT)^14^, Gaussian frameworks^15^, and K-nearest neighbors (KNN)^16,17^ are also explored for inspecting plant diseases. These ML-based approaches^18,19^ are easier to understand and require a small amount of data for model training, and, however, these approaches are time-consuming and highly rely on skilled human resources. Moreover, traditional ML-based feature extraction approaches always require a trade-off with respect to processing complexity and recognition effectiveness^20^.

To deal with ML-based approaches' limitations, DL-based methods are mainly tested^21^. Nowadays, several DL methods i.e. CNN^22^, Recurrent neural networks (RNNs)^23^, and long short term memory (LSTM)^24^ are highly evaluated in agricultural science. These approaches are proficient in robustly computing the descriptive set of image features without the need for human expertise. DL methods mimic the way the human brain works as people localize and recognize several objects by viewing their samples, and so do these methods to perform pattern recognition. DL-based frameworks show robust performance in the area of agricultural science and suit well to numerous tasks and various types of deep neural networks (DNNs) show better accuracy over hyperspectral analysis^25^. Techniques like GoogLeNet^26^, AlexNet^27^, VGG^28^ and ResNet^29^ are heavily explored in agriculture for tasks like measuring food quantity, identifying crop heads, fruit counting, crop disease recognition, and classification, etc. These methods are empowered to show effective classification results while reducing the computational time due to their ability to exploit the topological data from the input images^30^.

Although extensive work has been presented by researchers for tomato plant leaf diseases, its identification at the early stages is still a complex task due to the extensive chrominance similarity between the affected and unaffected plant regions^31^. Moreover, the varying plant leaf sizes, light and intensity variations, presence of noise, and blurring in the suspected images have further complicated the detection process. Therefore, there is still room for performance enhancement in terms of plant disease recognition accuracy and processing time. For this reason, we have modified a DL-based approach named Faster-RCNN where we have introduced the ResNet-34 framework as a feature extractor for tomato plant disease localization and categorization. The proposed work has improved the classification performance with the mAP and accuracy values of 0.981, and 99.97% respectively, and minimized the testing time with the value of 0.23 s. The proposed approach is capable of detecting and classifying the tomato plant leaf disease classification performance under the occurrence of several image artifacts like blurring, noise, light variations, etc. We have presented an extensive performance evaluation comparison with the latest approaches to show its robustness and have confirmed through experimentation that the proposed solution shows an efficient and effective solution to plant leaf disease classification.

## Related work

Here, we have presented an analysis of existing approaches employed in the field of agriculture to detect and classify numerous plant diseases.

Le et al.^32^ introduced a technique for classifying several plant leaf diseases. In the first step, a pre-processing step based on the morphological opening and closing methods was applied to eliminate the unwanted information from the suspected samples. After this, an approach namely the filtered local binary pattern method with contour mask and coefficient *k* (k-FLBPCM) was applied for keypoints extraction. The calculated feature vector was applied for the SVM classifier training to perform the classification. This work^32^ is robust to diagnosing plant leaf-related diseases with an accuracy of 98.63%, however, its performance decreases for distorted images. Similarly, a framework called Directional Local Quinary Patterns (DLQP) was presented in^33^ to calculate the descriptive set of sample features later employed for SVM training. The technique^33^ exhibits better performance for plant leaves abnormalities categorization with an accuracy of 97.80%, however, it shows degraded results for blurry input images. Sun et al.^34^ presented a solution for crop disease categorization. Initially, Simple Linear Iterative Cluster (SLIC) was used for dividing the input image into several blocks. Then, the fuzzy salient region contour and Gray Level Co-occurrence Matrix (GLCM) were applied for feature computation from the image blocks. The extracted keypoints vector was applied to train the SVM method to classify numerous plant diseases. The methodology^34^ demonstrates better plant leaf disease recognition results with an accuracy of 98.50%, however, it suffers from high computational cost. A similar method was introduced in^2^ where the GrabCut algorithm was used for image segmentation and the LBP method was used for extracting the sample features. Moreover, the SVM was trained over the extracted keypoints to categorize leaf abnormalities. The work presented in^2^ is effective for crop disease identification with an accuracy of 95%, however, it may not perform well for noisy images. Ramesh et al.^35^ presented a framework to achieve the classification of plant leaf diseases where the Histogram of Oriented Gradients (HOGs) approach was used to calculate the image features and the Random Forest (RF) classifier was used to perform the classification process. This approach presents a low-cost solution for plant leaf disease classification with an accuracy of 70.14%, however, it needs further classification performance enhancements. In^36^ a method was proposed to recognize the leaf diseases from the turmeric plant. Initially, the K-means technique was used for segmenting the suspected image sample. After this, the GLCM method was used for computing the image keypoints for the SVM training to execute the disease classification job. This technique^36^ works well for crop disease recognition with an accuracy of 91%, however, it is not robust to huge brightness changes in the suspected samples. An ML-based approach was introduced in^37^ for classifying several plant leaf diseases. Numerous techniques, namely, shift-invariant feature transform (SIFT), local binary pattern (LBP), and GLCM were applied to compute the keypoints of the suspected samples. The calculated features were exploited in the next step for training the SVM, KNN, and RF classifiers. This approach^37^ presents the best classification results with the RF classifier with an accuracy of 82.12%, however, the performance needs more improvements. In^38^ another framework was proposed to recognize plant diseases. In the first step, the fourteen color spaces were employed for computing the features from the input samples with a vector length of 172. Secondly, the extracted keypoints were utilized for the SVM classifier training with an accuracy of 94.65%. The method in^38^ demonstrates improved classification performance, however, it is unable to work well for the distorted samples.

Several researchers have explored the DL-based techniques for plant disease categorization and gained better results^39^. One of such works was presented in^40^ that employed a DL-based approach named as Few-Shot Learning (FSL) for locating the diseased area of plant leaves and specifying the associated class. For deep features computation, the Inception V3 method was applied while the SVM classifier was employed for the categorization task. The work presented^40^ shows better disease classification performance with an accuracy of 91.40%; however, the approach needs evaluation over a large and complex dataset. In^41^, a CNN-based network comprising of 3 convolution layers was proposed to calculate the image features and perform the classification task. This work gives a low-cost solution for the automated detection of crop diseases with an accuracy of 91.20%, however, it suffers from the problem of model over-fitting. Richey et al.^42^ proposed a lightweight plant disease detection approach that can be deployed to mobile phones. The technique was concerned to classify the maize crop disease. The ResNet50 framework was applied for feature computation and deciding the respective classes. The work demonstrated in^42^ performs well for plant disease recognition with an accuracy of 99%, however, not suitable for all types of cell phones due to the processing requirements.

Similarly in^43^, a work was proposed for tomato leaf disease identification. A deep feature extractor framework, AlexNet was used to compute the reliable set of image keypoints from the suspected sample which was later used to train the KNN classifier to perform the categorization job. The framework in^43^ is robust to tomato leaf disease classification with an accuracy of 76.10%, however, the KNN technique is a tedious and time-taking approach. Furthermore, another framework was presented in^44^ for tomato leaf disease classification where a residual framework was used for reliable keypoints extraction and a CNN-based approach was to categorize the diseased areas of plant leaves. The methodology in^44^ exhibits robust leaf disease recognition accuracy of 98%, however, at the expense of increased processing load. Dwivedi et al.^45^ presented a methodology named region-based CNN (RCNN) to recognize and categorize the several leaf diseases of grapes. Initially, the ResNet18 approach was used for calculating the reliable set of suspected image keypoints. The RCNN model later categorized the computed features into several classes. The work^45^ exhibits improved classification performance with a value of 99.93%, however, it exhibits lower performance to unseen examples. In^46^ several frameworks namely, VGG, ResNet, and DenseNet were used for feature computations from the input images. The work^46^ shows robust accuracy of 98.27% using the DenseNet approach, however, it suffers from a high economic burden. Although the research community has presented extensive work for the automated recognition and categorization of crop leaves contagious region categorization, however, still there is a need of performance improvement.

Saleem et al.^47^ employed several DL approaches like SSD, Faster-RCNN, and Region-based Fully Convolutional Networks (RFCN) to identify and categorize the diseased portions of plants. The work shows the highest mAP score of 73.07%. However, unable to identify the affected regions of very small sizes from the leaf samples. Zhao et al.^48^ proposed an approach to classify several tomato plant leaf diseases where the convolutional neural network that integrates an attention mechanism was applied. The approach acquired an accuracy value of 99.24%. Similarly in^30^ several DL-based approaches like AlexNet, GoogleNet, Inception V3, ResNet-18, and ResNet-50 were evaluated to categorize the tomato plant leaf samples into several classes. The work attains the highest results for the GoogleNet model with an accuracy of 99.39%. Moreover, Bhujel et al.^49^ proposed a DL-based approach namely the ResNet18 with CBAM for tomato plant leaf disease classification and acquires an accuracy of 99.69%. The works presented in^30,48,49^ improve the tomato plant leaf diseases classification performance, however, these methods perform the image level classification and are unable to locate the exact diseased region which is an important aspect for the automation of plant abnormality detection methods. To cover the limitations of such works, we have presented an object detection approach capable of detecting and classifying the diseased regions of tomato plant leaves with a small number of model parameters to reduce the computational complexity. Moreover, we are focused on presenting a model that can detect the affected areas of varying shapes and sizes.

A review of work from history used for the identification and categorization of several plants leaves abnormalities is presented in Table 1. Although the research community has presented extensive work for the automated recognition and categorization of crop leaves contagious region categorization, still there is a need for performance improvement.

**Table 1:** Review of existing techniques.

Reference	Technique	Limitation
**ML-based methods**		
^32^	K-FLBPCM with the SVM classifier	The work is not robust to distorted samples
^33^	DLQP with the SVM classifier	The work does not perform well with blurry images
^34^	GLCM with the SVM classifier	The approach is computationally complex
^2^	LBP with the SVM classifier	The method is not effective in noisy images
^35^	HOGs with the RF classifier	The method needs further performance enhancement
^36^	K-means + GLCM with the SVM classifier	The work is not robust to huge brightness changes in the suspected samples
^37^	SIFT, LBP, GLCM, along with the SVM, KNN, and RF classifiers	The work needs further classification accuracy improvements
^38^	Fourteen color spaces along with the SVM classifier	The work is not workable with distorted images
**DL-based methods**		
^40^	Inception V3 method along with the SVM classifier	The method requires evaluation over a large and complex dataset
^41^	CNN	The work is suffering from the model over-fitting
^42^	ResNet50	The work is computationally expensive
^43^	AlexNet along with the KNN classifier	The method is slow and time-consuming
^44^	CNN	The technique requires extensive data for model training
^45^	RCNN	The work is not generalized well to real-world examples
^46^	VGG, ResNet and DenseNet	The approach is economically inefficient
^47^	SSD, Faster-RCNN, RFCN	The work is unable to identify the affected regions of very small sizes
^48^	CNN with an attention mechanism	The work is computationally expensive and unable to locate the exact diseased region on the test sample
^30^	AlexNet, GoogleNet, Inception V3, ResNet-18, and ResNet-50	The work is computationally expensive and performs the image level classification
^49^	ResNet18 with CBAM	The method cannot identify the exact location of the diseased area from the suspected images

## Contributions

To cover the existing issues of tomato plant leaf disease classification, we have presented a DL-based approach named Faster-RCNN with ResNet-34 as a feature extractor for tomato plant disease localization and categorization. Following are the main contribution of our work:

- Modified an object detection technique namely Faster-RCNN for plant disease classification which enhanced the classification accuracy to 99.97%.
- We introduce a cost-effective approach to improve the tomato plant disease classification performance while reducing the testing time with a value of 0.23 s.
- Robust detection of the affected regions of plant leaves even under the existence of noise, blurring, color, size, and light alterations due to the efficacy of the Faster-RCNN technique to deal with the over-fitted training data.
- The presented framework is empowered to correctly localize the disease area of plant leaves with the mAP score of 0.981 because of the reliable feature extraction capability of the RESNET-34-based Faster-RCNN model.
- A thorough analysis has been conducted in comparison to other approaches to plant disease classification methods over a standard database namely PlantVillage to demonstrate the efficacy of the presented work.

## Materials and methods

**Figure 1:**
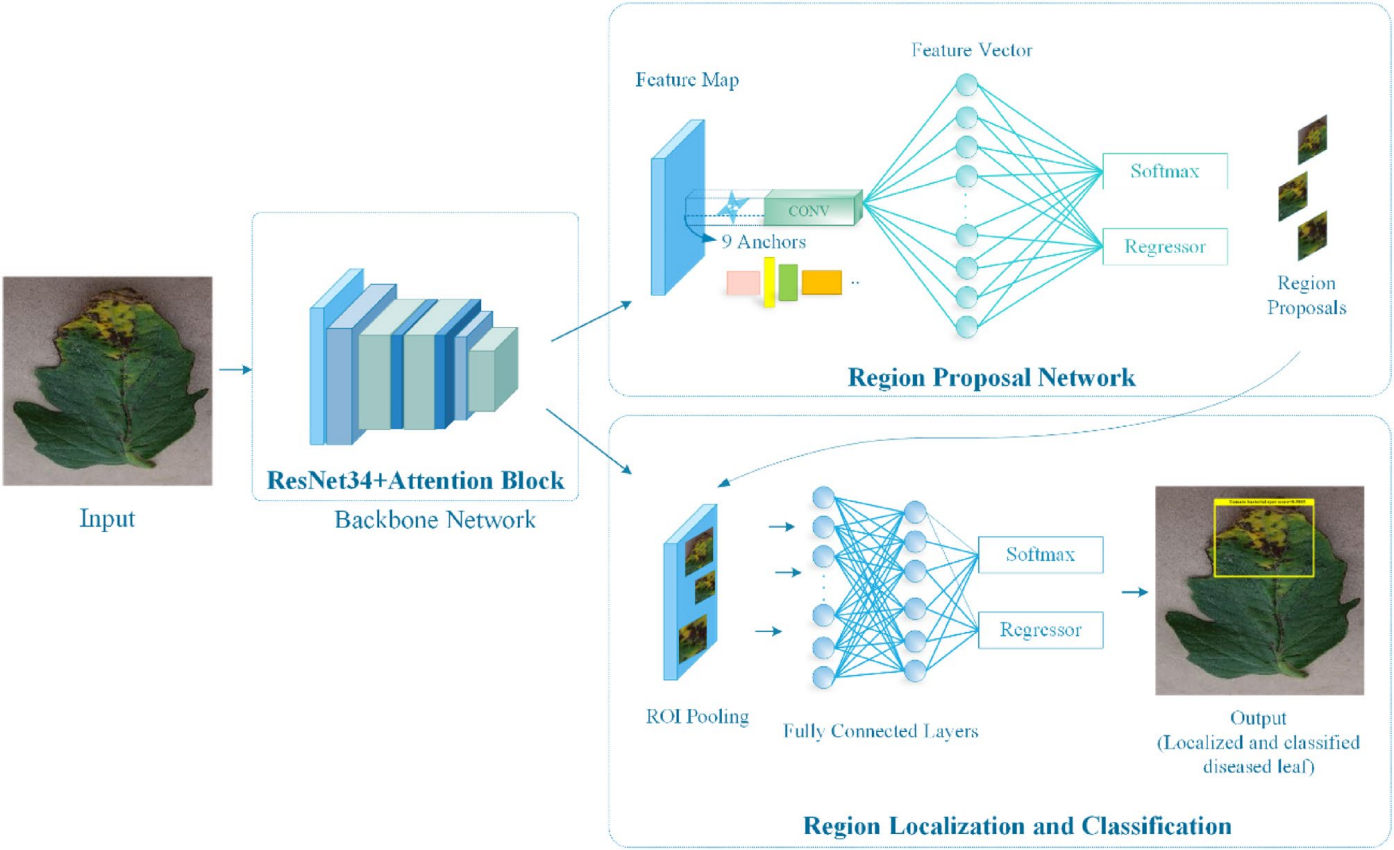
Workflow representation of the introduced approach.

In the presented framework, we have introduced an approach for identifying and categorizing several tomato plant leaf diseases from the suspected images via employing a DL-based approach namely the Faster-RCNN with the ResNet-34 as the base network. Initially, annotations are created to specify the affected region from the input images. The annotated samples are passed to the Faster-RCNN framework where the ResNet-34 base network computes the deep features which are localized and classified by the Faster-RCNN framework. Finally, the trained model is evaluated on the test data to identify and classify several tomato plant diseases. The entire workflow of the introduced solution is exhibited in Fig. 1. Our evaluation results confirm that Faster-RCNN with ResNet-34 feature extractor is robust to tomato plant disease classification due to its high recall ability.

### Material

To assess the classification power of the introduced solution, we have used the PlantVillage dataset^50^, a standard and freely available database. The PlantVillage dataset contains a total of 54,306 samples from 14 types of plants. We have considered the images of tomato plants from the PlantVillage dataset with 10 specified classes for our work. The main motivation for using the PlantVillage database is that it contains images with intense changes in the dimensions, color, and location of the diseased areas.

**Figure 2:**
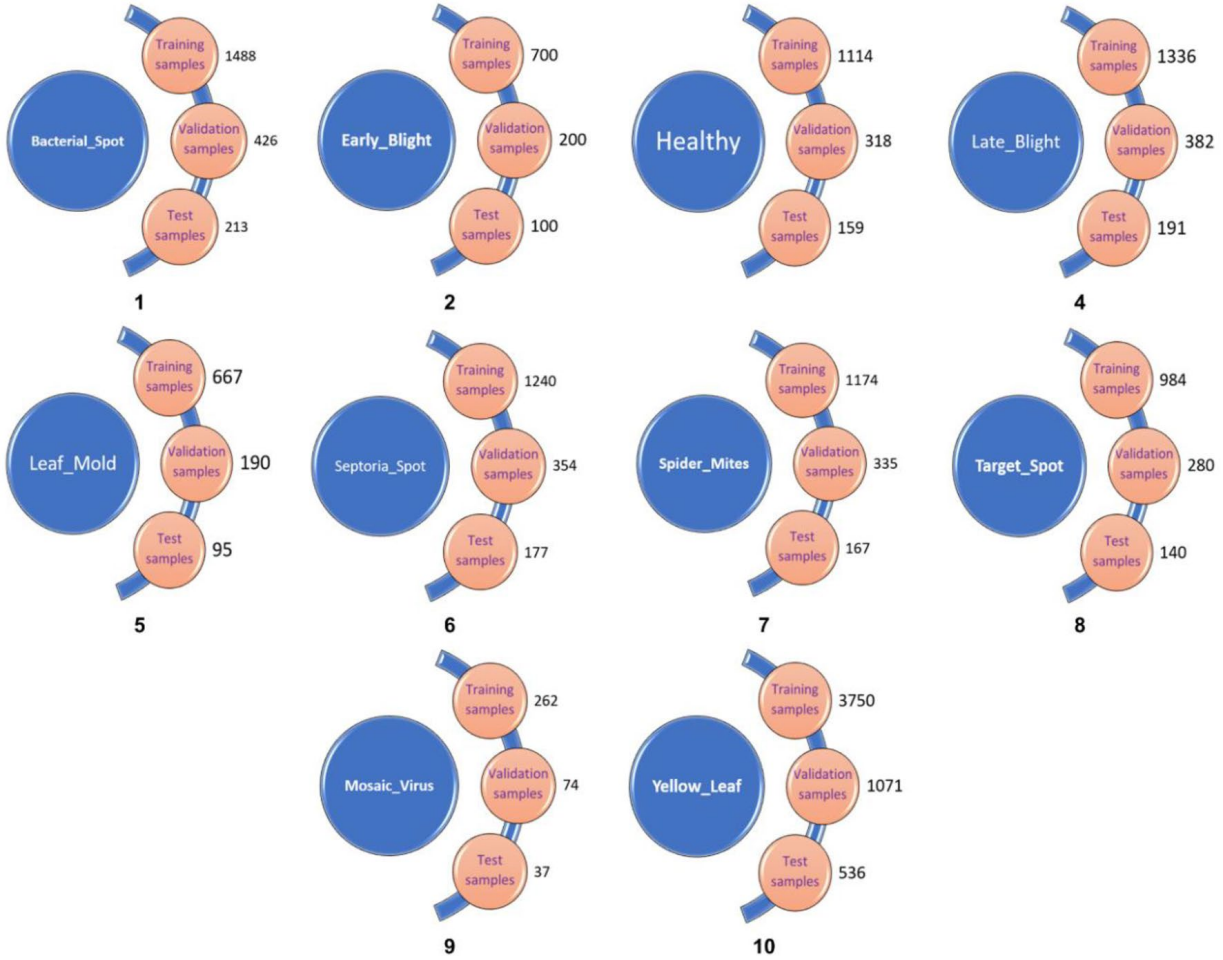
Detailed description of the employed dataset.

**Figure 3:**
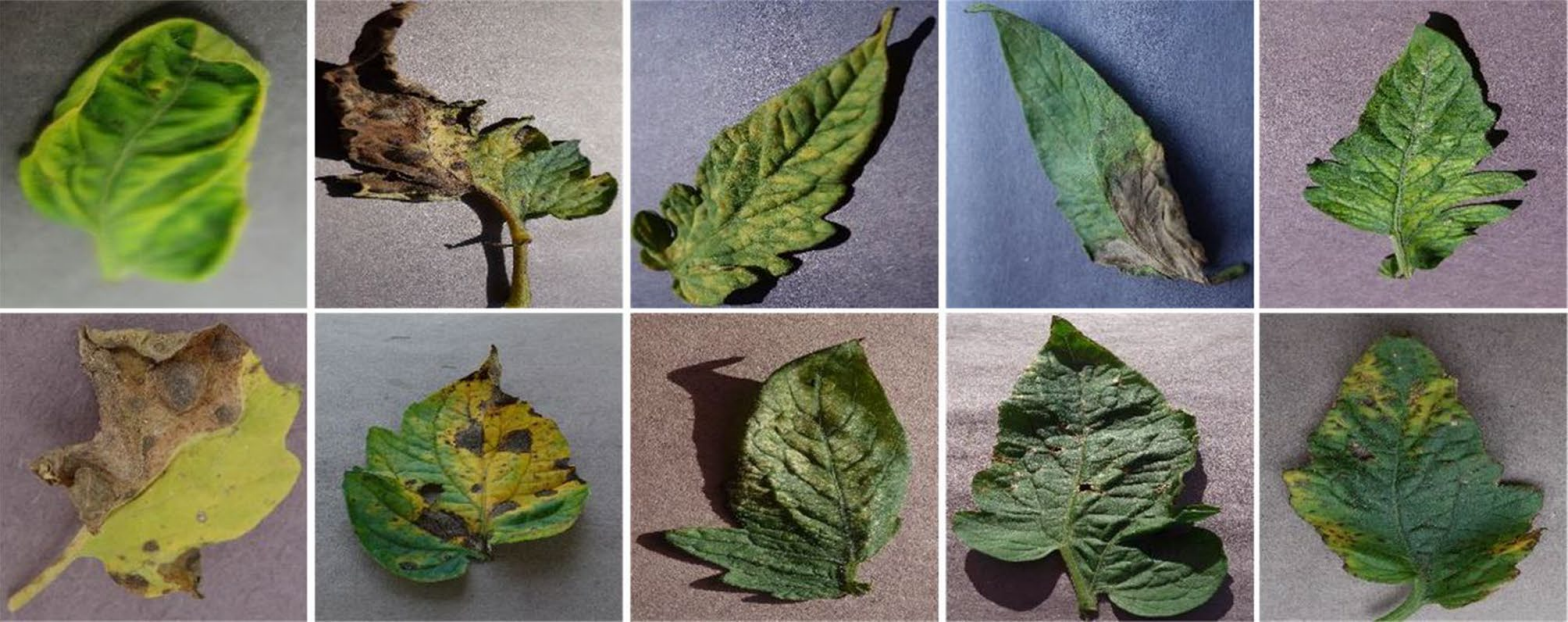
Samples of the PlantVillage dataset.

Moreover, samples are suffering from noise, blurring, and light intensity variations. A detailed description of tomato images is given in Fig. 2 while a few samples are shown in Fig. 3.

### Ground truth generation

**Figure 4:**
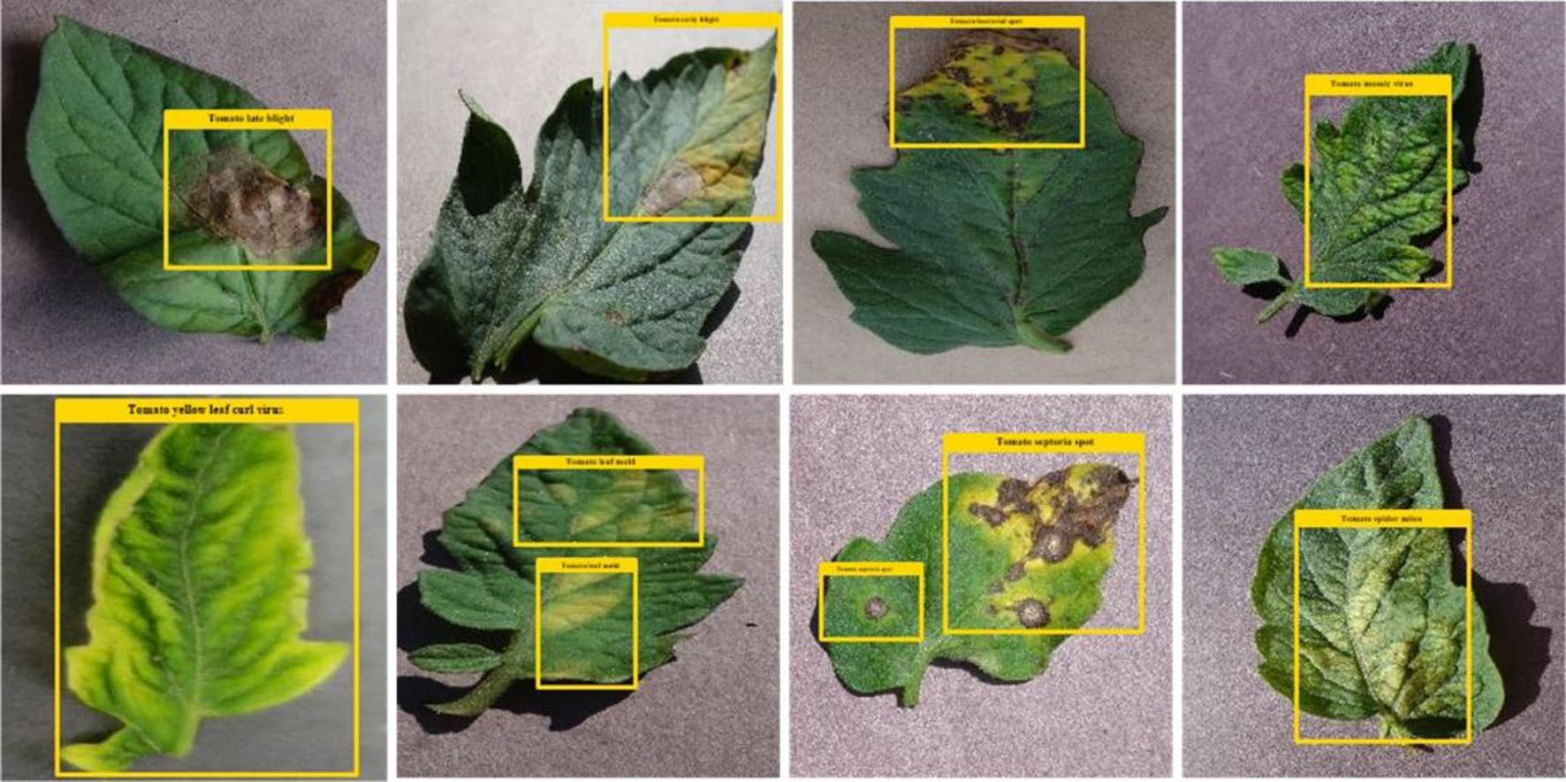
Annotated image samples.

To have a robust and accurate training procedure, it is essential to determine the affected portion from the suspected samples correctly. To accomplish this, we have used LabelImg^41^, an open-source software to develop plant images' annotations to locate the RoIs. The final output containing the coordinate values of affected regions is saved in a CSV file which is later passed along with the input images to the Faster-RCNN framework for model training. Some examples of annotated samples are exhibited in Fig. 4.

### Faster-RCNN

In the introduced framework, we have employed Faster-RCNN, a DL-based method for the automated localization and categorization of several plant leaf abnormalities. The Faster-RCNN approach utilizes the convolve-filters which gives it the ability to evaluate the structure of the suspected sample and extract a reliable set of key points from it. We have selected the Faster-RCNN framework over the RCNN and Fast-RCNN models for tomato plant disease classification as these methods are computationally more complex. Moreover, RCNN and Fast-RCNN models employ handcrafted approaches i.e. EdgeBox^51^ or selective search^52^, etc. for feature computation which in turn is unable to learn a discriminative set of image features. Whereas, the Faster-RCNN approach robustly resolves the issues of the RCNN and the Fast-RCNN approaches via introducing a separate module namely the Regional Proposal Network (RPN) for the automated extraction of features from the input image.

In the area of crop leaves disease localization and classification, identifying the ROIs from the suspected samples are suffering from two main challenges: (1) Exactly identifying the affected regions on the leaves since there is a huge color similarity between affected and healthy regions. (2) Correctly identifying the class associated with each identified region.

The selected framework namely Faster-RCNN can better tackle the above-mentioned challenges. As, the RPN module of Faster-RCNN has enabled it to better locate the ROIs and effectively classify the suspected samples as it employs the information of diseased portion size, color, and texture and guarantees a higher recall rate via using a few selected windows.

### Custom faster-RCNN

The conventional Faster-RCNN framework employed either VGG-16 or ResNet-101 as the base networks to calculate the reliable keypoints of the suspected sample. However, both these models comprise an extensive number of model parameters which in turn increase the computational cost of the Faster-RCNN. Moreover, such structure of the Faster-RCNN results in the vanishing gradient issue. To tackle the existing problems of the Faster-RCNN model, we have presented a modified version by presenting the ResNet-34 along with the CBAM as the feature extraction module. The presented ResNet-34 approach contains fewer model parameters, giving it a computational advantage over the existing base networks. The detailed demonstration of the trainable parameters used for the proposed method is explained in Table 2. The introduced work was implemented in Python language with Tensorflow and Keras libraries and executed on an Nvidia GTX1070 GPU-based system in Windows 10 environment.

**Table 2:** Details of trainable parameters used by the introduced work.

Network parameters	Value
Total epochs	20
Learning rate	0.001
Employed batch size	8
Value of threshold for the matched region	0.2
Value of threshold for the unmatched areas	0.5

The Faster-RCNN approach comprises four steps: feature extraction module, Region proposal networks (RPN), RoI pooling, and classification to locate and classify several plant diseases.

### ResNet-34 based features extractor

**Figure 5:**
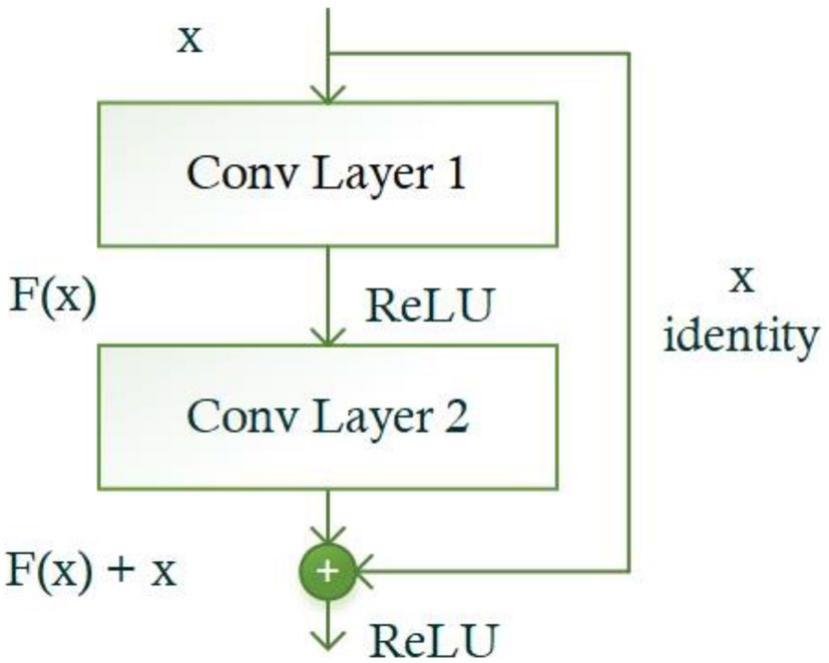
The architectural representation of the basic building block ‘Residual Block’ in ResNet.

A backbone network is usually a CNN model that is responsible for extracting feature maps that present semantic and robust representations of an input image. These feature maps are used for detecting regions of interest and classification in several object detection algorithms. Intuitively, the more robust the computed features of the backbone network are, the higher the detection performance^53^. In this study, we employed ResNet-34^54^ along with Convolutional Block Attention Module (CBAM)^55^ for improved feature extraction in the proposed Faster RCNN model, which ultimately improved the classification performance for plant disease identification tasks. ResNet^54^ is the latest CNN model that utilizes identity shortcut connections and residual mapping across layers to achieve high accuracy. In typical deeper networks, each layer feeds its output into the next layer and thus can compute more complex features that improve the model's robustness and performance^56^. However, in these networks, performance abruptly degrades with the increase in the network depth due to gradient vanishing problems during training. To address this issue, the ResNet model introduced skip connections in deep networks that bypass one or more layers and form the basis for residual blocks. The resulting architecture allows reusing the feature maps from the preceding layers, which provides better accuracy and easier training. The detailed structure of the ResNet-34 model is presented in Table 3. The model contains 33 convolutions (Conv) layers in total, which are grouped into 5 Conv stages, each consisting of several residual blocks placed on top of one another. Figure 5 depicts the structure details of the individual residual blocks. A residual block comprises several Conv layers, a ReLU activation method, a batch normalization layer, and a shortcut connection. In residual block, the stacked layers conduct residual mapping by establishing shortcut connections that perform locating mapping (x). Their results are combined with the stacked layers' output residual function F (x). The resultant value of the residual block can be expressed as:

**Table 3:** Architecture details of ResNet-34 original and modified.

Layer name	Original	Modified
Conv1	7 × 7, 64, 3 × 3 max pool	$\left[3\times 3, 64\right]$× 3, 7 × 7 Attention
Conv2_x	$$\left[\begin{array}{c}3\times 3, 64\\ 3\times 3, 64\end{array}\right]\times\,3$$	$$\left[\begin{array}{c}3\times 3, 64\\ 3\times 3, 64\end{array}\right]\times\,3$$
Conv3_x	$$\left[\begin{array}{c}3\times 3, 64\\ 3\times 3, 64\end{array}\right]\times\,4$$	$$\left[\begin{array}{c}3\times 3, 64\\ 3\times 3, 64\end{array}\right]\times\,4$$
Conv4_x	$$\left[\begin{array}{c}3\times 3, 64\\ 3\times 3, 64\end{array}\right]\times\,6$$	$$\left[\begin{array}{c}3\times 3, 64\\ 3\times 3, 64\end{array}\right]\times\,6$$
Conv5_x	$$\left[\begin{array}{c}3\times 3, 64\\ 3\times 3, 64\end{array}\right]\times\,3$$	$$\left[\begin{array}{c}3\times 3, 64\\ 3\times 3, 64\end{array}\right]\times\,3$$

1 $$Z=F(x)+x$$

Here, x is the input, F represents the residual function and Z is the output of the residual function.

We modified the architecture of ResNet-34 by adding CBAM based attention block^55^ at the initial of the network. The motivation to add the CBAM block is that it improves the representation of features by employing an attention mechanism. The attention block assists the network to focus on the disease-effected locations while suppressing irrelevant background information and improving recognition performance under varying challenging conditions, such s color, light, and intensity variations. The CBAM module refines the CNN-derived features by integrating spatial and channel-wise attention and thus enhances DNN performance. Due to its lightweight architecture, the CBAM block adds a negligible overhead and can be trained end-to-end along with base CNNs. Table 3 presents the architecture of a modified backbone network with an attention block. We further replaced the early 7 × 7 Conv and max-pooling layer with three stacked 3 × 3 Conv layers to avoid downsampling steps in the early convolutional layer^57^. Furthermore, to decrease computational costs, the channel for newly added Conv layers is set to 64.

### Region proposal networks (RPN)

The RPN unit comprises 3 × 3 convolutional layers which create the anchors and bounding boxes from which object proposals are computed.

### RoI pooling

The ROI pooling layer employs the result of both the convolution layers and the RPN module to calculate the proposal feature maps which are later passed as input to all fully connected layers. This phase includes employing the result of the preceding two layers by utilizing the feature map and object proposals to compute the proposal feature maps which are later fed to fully connected layers.

### Classification

In the last step, the Faster-RCNN module performs localization and classification by generating the bounding boxes to identify the diseased region of the tomato crop and determine the associated category.

The detailed steps of the proposed solution are presented in Algorithm 1.

## Results

In this part, we have discussed the description of the employed dataset and explained the obtained results in detail.

### Evaluation metrics

**Figure 6:**
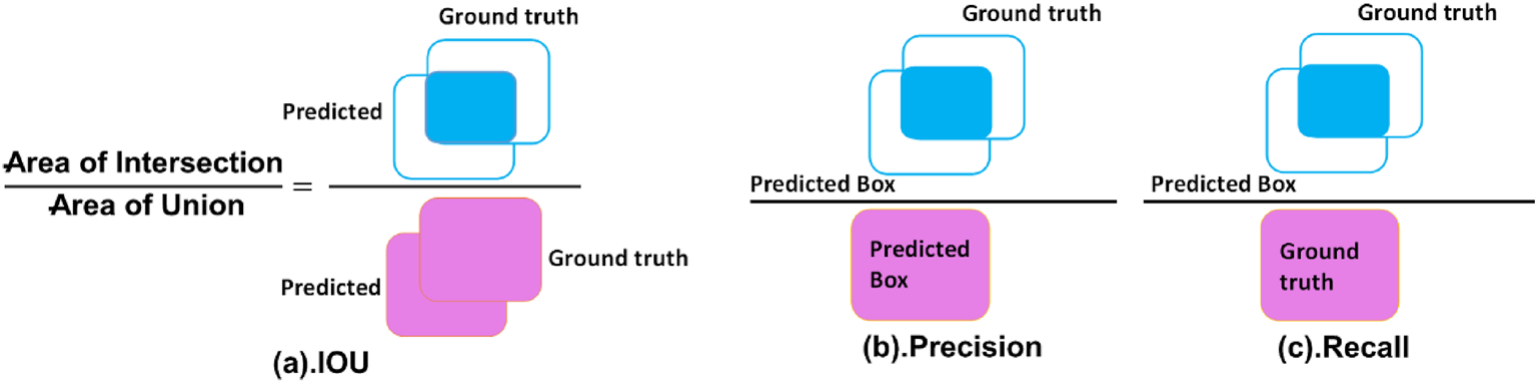
Visual demonstration of IOU, precision, and recall.

The evaluation metrics namely accuracy, mean average precision (mAP), intersection over union (IOU), precision, and recall are used to evaluate the robustness of our work which are defined in Eqs. (2) and (3) and Fig. 6, respectively.

2 $$Accuracy = \frac{TP + TN}{{TP + FP + TN + FN}}$$

3 $$mAP: = \sum\limits_{i = 1}^{T} {AP(t_{i} )/T}$$

In Eq. (3), *AP* denotes the average precision of each class and *t* is the query or test image. *T* is the total number of test samples.

### Localization results

**Figure 7:**
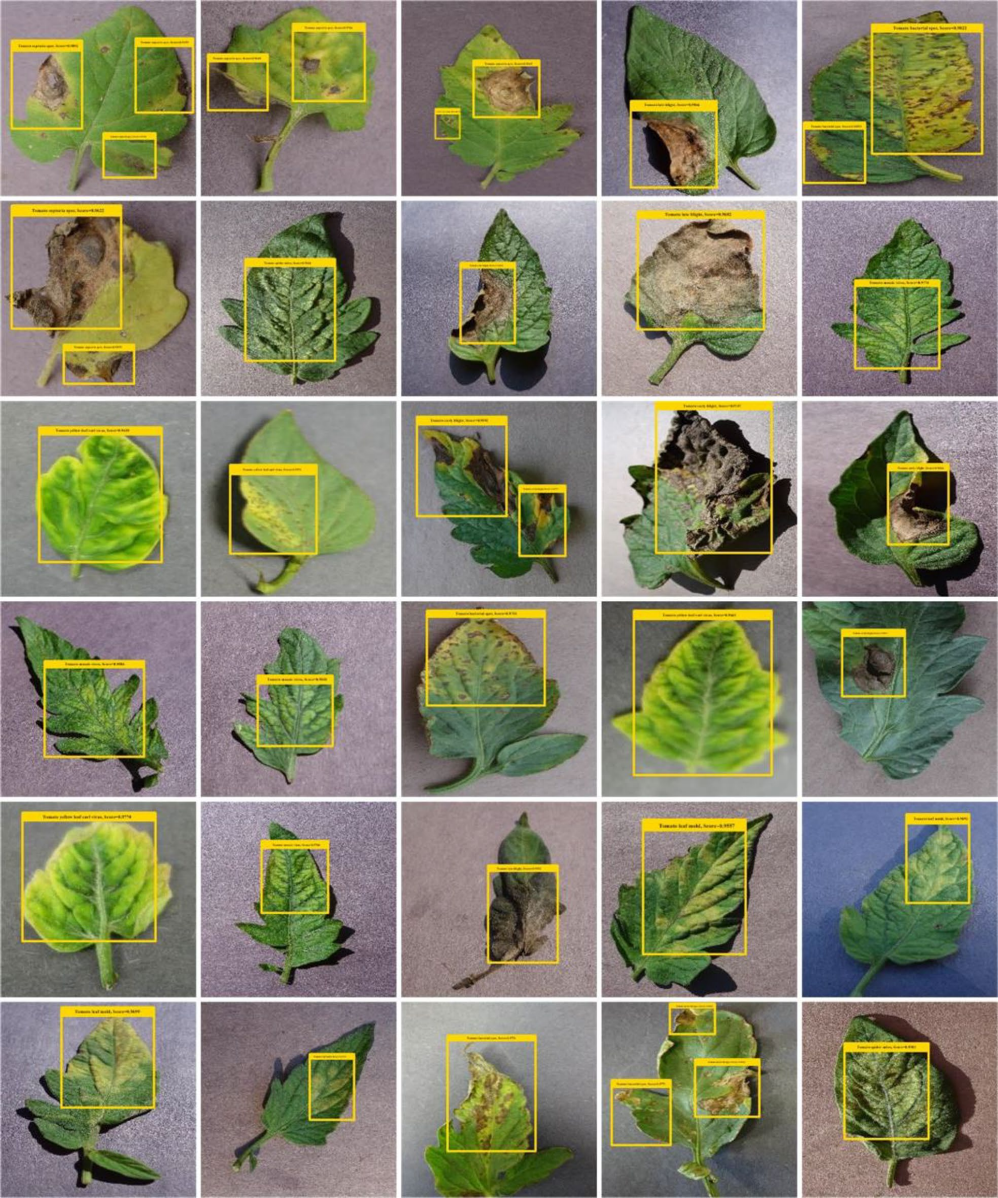
Localization results.


The main characteristic of an automated plant disease system is that it should be capable of localizing the various categories of plant diseases accurately. Therefore, to evaluate the detection performance of the introduced solution, the tomato images from the employed database and show some of the visual results in Fig. 7.

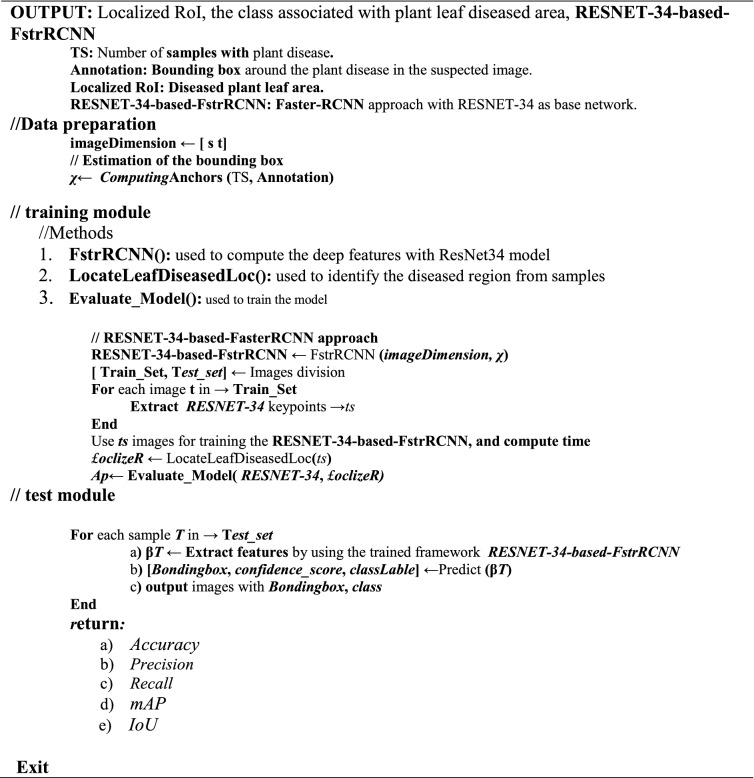


From the reported results, it can be analyzed that the presented approach is robust to classify the samples of varying classes under the occurrence of several image transformations like under the presence of leaf size, color, and diseased portion position variations.

The recognition ability of the Faster-RCNN model allows it to locate and categorize numerous tomato plant leaf diseases correctly. To numerically demonstrate the localization supremacy of the proposed method, two standard metrics namely mAP and IOU are explored. These measures assist to check the detection and classification enactment of the technique for numerous categories of tomato plant anomalies. More specifically, we obtain the mAP score of 0.981and IOU values of 0.973, respectively. It is quite visible from the results that our presented framework can be consistently used to identify and categorize tomato plant diseases.

### Classification results

**Figure 8:**
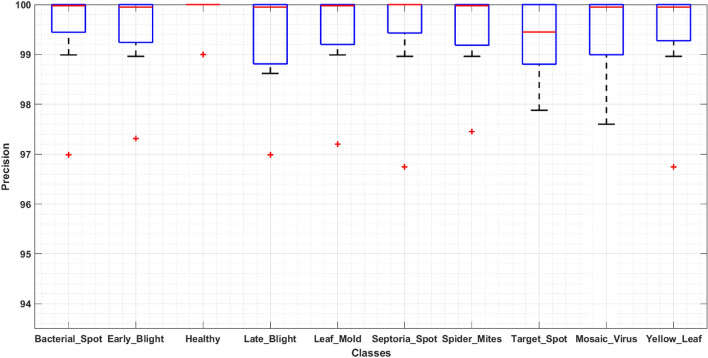
Evaluation using Precision.

**Figure 9:**
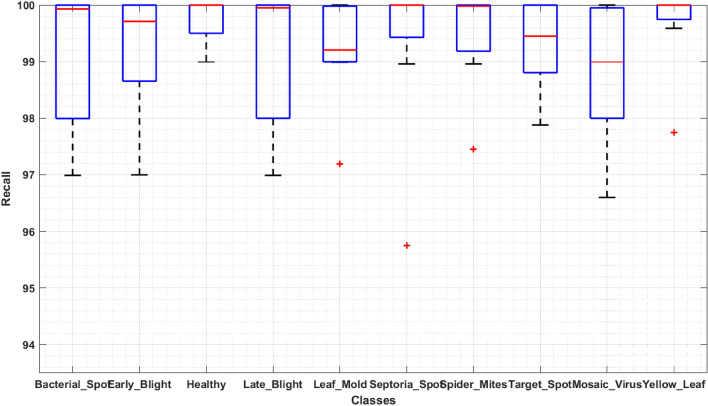
Proposed method evaluation using Recall.

To design an accurate and robust tomato plant leaf disease classification model, it should be able to differentiate between the various classes of plant abnormalities. For this reason, we have designed an experiment to discuss the classification results of our approach in detail. In this section, we have demonstrated the category-wise identification and classification results of ResNet-34-based Faster-RCNN in the form of precision, recall, F1-score, and an error rate. To evaluate the classification performance of the proposed ResNet-34-based Faster-RCNN on ten classes, we have drawn the boxplots (Figs. 8, 9) to show the obtained precision and recall rates as these plots have the ability to better show the results by exhibiting the minimum, maximum, median values along with the symmetry and skewness of the data. Figures clearly show that our technique can detect and classify the different types of tomato plant leaf diseases.

**Figure 10:**
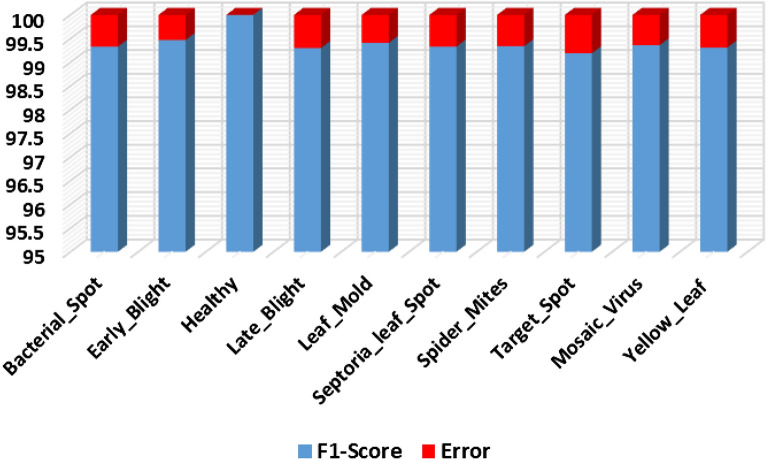
Evaluation using F1 score and error rate.

Furthermore, the F1-score and the error rate for the entire dataset is shown in Fig. 10. More clearly, we have obtained an average F1-score of 99.42% along with the minimum and maximum error rates of 0% and 0.35%, respectively.

It is quite evident from the reported results that our approach has better recall ability and can better generalize to tomato plant diseases of several types.

**Figure 11:**
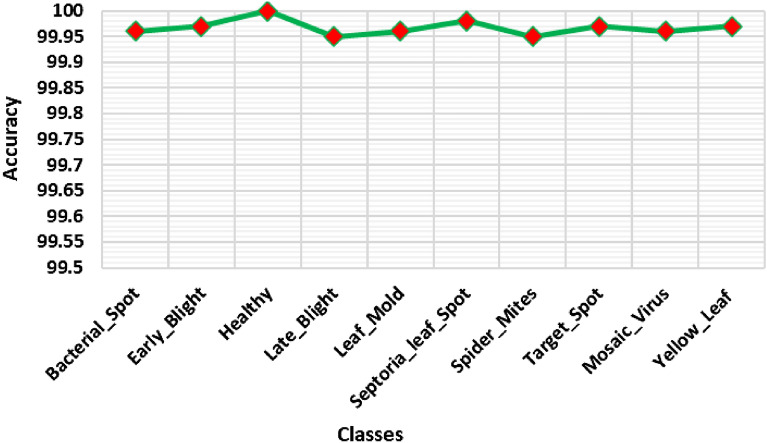
Line plot of accuracy measure.

To further elaborate on the recognition accuracy of our approach, we have analyzed to show the class-wise classification performance of our approach, and the results are shown in Fig. 11. More clearly, for all the ten classes of tomato plant leaf diseases from the PlantVillage dataset, the ResNet-34-based Faster-RCNN approach acquires the average accuracies of 99.96%, 99.97%, 100%, 99.95%, 99.96%, 99.98%, 99.95%, 99.97%, 99.96%, and 99.97% respectively which is showing the robustness of our approach with high recall rate.

**Figure 12:**
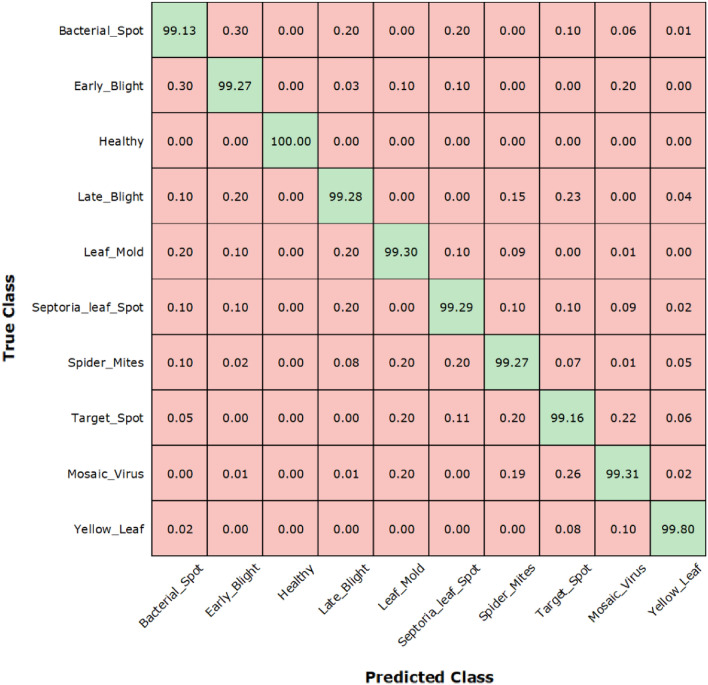
Confusion matrix of our method.

We further demonstrate the obtained results for each class of tomato plant leaf disease, we have shown the confusion matrix (Fig. 12) as it better explains the results by showing the real and predicted class. Figure 12 clearly shows that the presented custom Faster-RCNN can effectively differentiate all classes of tomato plant diseases. So, from the entire result discussion, it can be concluded that the introduced methodology is more effective to both tomato plant leaf diseases portion identification and categorization.

### Comparison with base approaches

We have analyzed the robustness of our framework against several DL-based models namely GoogleNet, ResNet-101, Xception, VGG-19, and SE-ResNet50 via an experiment. The obtained results are shown in Table 4. From the reported results in Table 4, it can be viewed that our approach outperforms the base techniques. More specifically, the GoogleNet approach shows the minimum results with the average precision, recall, F1-score, and accuracy values of 87.16%, 87.09%, 87.12%, and 87.27% respectively. While the second minimum values are attained by the Xception model with the average precision, recall, F1-score, and accuracy values of 88.25%, 88.14%, 88.19%, and 88.16% respectively. While in comparison, the proposed improved Faster-RCNN approach with ResNet-34 shows the highest performance with the average precision, recall, F1-score, and accuracy values of 99.48%, 99.32%, 99.42%, and 99.97%, respectively. More clearly, in the case of precision metrics, the comparative base models show an average value of 90.50%, which is 99.48% for our method, so we have attained a performance gain of 8.97%. While for the recall metric, the comparative approaches show an average value of 90.53%, while our method shows an average recall value of 99.42%. Therefore, in the case of the recall evaluation metric, we have shown an average performance gain of 8.89%. Moreover, for the F1-Score and accuracy metrics, the base models show the average values of 90.91%, and 90.56% respectively, while the proposed solution shows the average values of 99.42%, and 99.97% respectively. So, for the F1-Score and accuracy metrics, the proposed solutions show performance gains of 8.51%, and 9.41% respectively. From the performed analysis, it can be concluded that our work is more effective for both tomato plant leaf disease detection and classification. The major cause of the efficient classification accuracy of the proposed approach is that it uses the ResNet-34 model with CBAM as the feature extractor module which extracts a more robust set of image keypoints by removing the redundant data and minimizing the network complexity which in turn makes the model prone to model overfitting problems.

**Table 4:** Comparative analysis of our approach with the base models.

Model	Precision (%)	Recall (%)	F1-score (%)	Accuracy (%)
GoogleNet	87.16	87.09	87.12	87.27
ResNet-101	89.95	90.13	90.04	90.13
Xception	88.25	88.14	88.19	88.16
VGG-19	90.39	90.47	92.43	90.42
SE-ResNet50	96.77	96.81	96.79	96.81
Proposed	99.48	99.38	99.42	99.97

### Comparative evaluation with DL-based methods

We performed an analysis to compare the performance of the presented approach with other object detection techniques employed for the plant leaf disease classification. All employed approaches have been evaluated for several situations, i.e., a case where several plant leaves are present in a single suspected sample or to check their performance for different plant classes. The main reason to perform this simulation is to check how accurate our system is compared to other DL-based techniques in locating the diseased leaf portion from the healthy parts of image leaves and differentiating abnormalities of the several plant types.

For this reason, we have selected the approaches namely Fast-RCNN^58^, Faster-RCNN^59^. You Only Look Once (YOLO)^60^, and single-shot detector (SSD)^61^. To perform the evaluation, we have chosen the mAP evaluation metric, a standard and most widely employed performance measure for object detection techniques by the research community. The results are shown in Table 5, clearly demonstrating that our technique has acquired the highest mAP value of 0.981. The SSD approach exhibits a lower mAP value of 0.830. While the second-lowest performance is shown by the YOLOv3 model with the mAP value of 0.830. As, these both methods are unable to locate the diseased portion of small sizes, therefore their detection accuracy dropped for such scenarios.

**Table 5:** Comparative analysis of the presented method DL-based approaches.

Models	mAP
Fast-RCNN	0.860
Faster-RCNN	0.884
YOLOv3	0.842
SSD	0.830
Proposed Faster-RCNN (RESNET-34)	0.981

While the Faster-RCNN with the base VGG-16 network shows better results with the mAP value of 0.884, this approach is computationally inefficient due to its extensive number of model parameters. On the other hand, the proposed method, ResNet-34-based Faster-RCNN has better tackled the limitations of the comparative methods as its feature extractor allows it to better compute the image features which allows it to learn the image transformations better. More specifically, the comparative DL-based object detection models give an average mAP value of 0.854, which is 0.981 in our case, so our method gives an average performance gain of 12.7%. Furthermore, the proposed Faster-RCNN contains fewer model parameters compared to other approaches, which also gives it a computational advantage.

### Proposed work evaluation with the state-of-the-art methods

To further measure the detection performance of our approach, we have taken the latest approaches^30,48,49,62–64^ and compared our results with them. We have used three metrics for performance analysis, precision, recall, and accuracy, and comparison evaluations reported in Table 6. It can be seen from Table 6 that our work performs well than the comparative approaches. The reason for the efficient performance of our approach is that the methods^30,48,49,62–64^ employ very complex network architectures which result in model overtraining. Whereas the proposed solution deploys a shallow network architecture as it uses the ResNet-34 model with CBAM which is more empowered to compute the more distinguishing set of image keypoints which assists in attaining robust performance. Moreover, the lightweight architecture of the model further assists to avoid the occurrence of the model overfitting problem. Such architecture of the proposed approach assists in better tackling the image transformation issues like the variations in the light, color, size, and position of leaves and improves the accuracy of tomato plant disease classification.

**Table 6:** Performance analysis with the latest approaches.

Approach	Precision (%)	Recall (%)	Accuracy (%)
Agarwal et al.^62^	90	92	91.20
Tm et al.^63^	94.81	94.78	94
Kaur et al.^64^	98.8	98.80	98.80
Zhao et al.^48^	–	99.24	99.24
Gutiérrez et al.^30^	99.29	99.12	99.39
Bhujel et al.^49^	–	–	99.69
Proposed	99.48	99.38	99.97

## Conclusions

The manual detection and classification of the various plant leaf diseases require the expertise of humans to identify the small details from the suspected samples. Furthermore, the intense variations in the size, color, and structure of plants further complicate the categorization procedure. To deal with such challenges, we have proposed a robust approach namely ResNet-34 based Faster-RCNN to detect and classify the diseases of tomato plant leaves. More specifically, we employed the ResNet-34 along with the CBAM as the base backbone of the Faster-RCNN model to compute the deep features from the input samples. Then, the extracted features are used to train the Faster-RCNN framework to locate and categorize the suspected samples into ten related classes. The results are evaluated on the tomato leaf diseased images taken from a challenging dataset, the PlantVillage database. Both the qualitative and quantitative results confirm that the presented solution is robust to plant leaf disease detection which can replace the manual systems. Moreover, the proposed method shows a low-cost solution to tomato leaf disease classification which is robust to several image transformations like the variations in the size, color, and orientation of the leaf diseased portion. Furthermore, the framework can locate the affected plant leaves under the occurrence of blurring, noise, chrominance, and brightness variations. As the existing work is concerned to locate the disease portion from the plant leaves only, therefore, in the future, we plan to extend our approach to apply it to other parts of plants as well.

## Data Availability

The data generated to support the findings of this study are available from the corresponding author upon reasonable request.
